# IMP321 (sLAG-3), an immunopotentiator for T cell responses against a HBsAg antigen in healthy adults: a single blind randomised controlled phase I study

**DOI:** 10.1186/1476-8518-5-5

**Published:** 2007-03-29

**Authors:** Chrystelle Brignone, Caroline Grygar, Manon Marcu, Gaëlle Perrin, Frédéric Triebel

**Affiliations:** 1 Immutep S.A., Parc Club Orsay, 2 rue Jean Rostand 91893, Orsay, France

## Abstract

**Background:**

LAG-3 (CD223) is a natural high affinity ligand for MHC class II. The soluble form (sLAG-3) induces maturation of monocyte-derived dendritic cells *in vitro *and is used as a potent Th1-like immune enhancer with many antigens in animal models. To extend this observation to human, a proof of concept study was conducted with a clinical-grade sLAG-3, termed IMP321, coinjected with alum-non-absorbed recombinant hepatitis B surface antigen.

**Methods:**

In a randomised, single blind controlled phase I dose escalation study, 48 seronegative healthy volunteers aged 18–55 years were vaccinated at 0, 4 and 8 weeks by subcutaneous injection with 10 *μ*g HBsAg mixed with saline (control) or with IMP321 at one of four doses (3, 10, 30 and 100 μg). To evaluate the efficacy of this three injections over 2 months immunization protocol, an additional control group was injected with the commercial vaccine Engerix-B^®^.

**Results:**

IMP321 was very well tolerated. Indeed, a lower incidence of adverse events was reported from the HBsAg plus IMP321 groups than from the Engerix-B^® ^group. HBsAg-specific antibody responses (anti-HBs) appeared sooner and were higher at 8 and 12 weeks in IMP321 recipients compared to HBsAg control subjects. More importantly, increased numbers of responders to HBsAg were found in IMP321 recipients compared HBsAg group, as revealed by higher post-vaccination frequencies of CD4 Th1 or CD8 Tc1 antigen specific T cells. IMP321 induced CD4 Th1 antigen-specific T cells in some of these naïve individuals after only one injection, especially in the 10 and 30 μg dose groups.

**Conclusion:**

IMP321 as an adjuvant to HBsAg was well-tolerated and enhanced T cell response vaccine immunogenicity (i.e. induced both CD4 Th1 and CD8 Tc1 antigen-specific T cells). This latter property has allowed the development of IMP321 as an immunopotentiator for therapeutic vaccines.

## Background

A clinically effective therapeutic vaccine to fight viruses or tumour requires the generation and expansion of specific cytotoxic T lymphocytes (CTL) able to proliferate and/or secrete Th1-type cytokines such as IL-2, IFNγ or TNF-α after antigen-specific stimulation. Since few years, many efforts have been done to attempt to amplify the immune response and to shift it towards an adequate response using adjuvants. Almost all therapeutic vaccine adjuvant approaches use ligands for one of the Toll-like receptors (TLR) expressed on DC. The most studied of the TLR ligands are the TLR9 ligands deoxycytidyl-deoxyguanosin oligodeoxynucleotides (CpG ODNs) or immunostimulatory DNA sequences (ISS) that are potent inducers of inflammation ("danger signals").

In addition to the TLR agonists that are *innate *immunity ligands, the immune response involves two *adaptive *immunity ligands that are expressed on activated T cells and bind to non-TLR receptors expressed on DC. These are the CD40L and lymphocyte activation gene-3 (LAG-3 or CD223) human proteins. Soluble forms have been tested at the preclinical and/or clinical stage as vaccine immunological adjuvants. Clinical development of soluble CD40L (sCD40L) has been hampered by an increased risk of thrombosis due to direct platelet activation by sCD40L [1]. Soluble LAG-3 (sLAG-3) binds to MHC class II molecules and induces dendritic cells (DC) to mature and migrate to secondary lymphoid organs where they can prime naïve CD4-helper and CD8-cytotoxic T cells [2-4], leading to tumour rejection [5-7]. This maturation effect is obtained specifically with sLAG-3 but not with any of the tested MHC class II mAbs [3], and is dependent upon the specific binding of sLAG-3 to MHC class II molecules located in membrane lipid raft microdomains [8]. Finally, the immunostimulatory activity of sLAG-3 in inducing tumour-associated human antigen-specific CD8^+ ^T cell responses to a much greater extent than CpG ODN [9] has been reported recently [10], further supporting the use of this recombinant protein as a promising candidate adjuvant for cancer vaccination.

In the present study, we report on the clinical and biological effects, and safety evaluation of IMP321, a GMP-grade sLAG-3 (hLAG-3Ig) protein, in a large randomised single blind phase I clinical trial. The results of this proof-of-concept clinical study in healthy volunteers using HBsAg as a model antigen has paved the way for the development of this human protein as an immunopotentiator for therapeutic vaccines.

## Methods

### Study design and subject selection

This single blind controlled phase I study was conducted at the Aster-Cephac S.A. facility in Paris. Ethical Review Board approval was obtained and each patient provided voluntary informed consent. Eligible subjects were healthy adult HBV vaccine naïve volunteers, aged 18–55, with no serologic evidence of previously resolved or current HBV infection. However, three of these were later found to be seroconverted (but not seroprotected) at baseline in the post study HBsAg antibodies determination (subjects #019, 035 and 044). Other exclusion criteria included liver enzyme levels outside the normal range, chronic HIV or HCV infection, or evidence of any other clinically significant acute or chronic disease. Subjects receiving immune suppressive medication, and those diagnosed with an immune or autoimmune dysfunction were not considered for this study. Female subjects had to have gone through the menopause for a least one year, as evidenced by lack of menstruation for the last 12 months and hormones (FSH, estradiol) blood level measurement at screening confirming menopausal status.

### Vaccines

For the production of a clinical batch of IMP321, CHO DHFR^- ^cells were transfected with a plasmid coding for the D1-D4 extra-cellular domains of human LAG-3 fused to the Fc tail of a human IgG1 [11]. A production clone was selected after amplification in methothrexate. The final container clinical batch used in the present study has a concentration of 1.1 mg/ml IMP321 (a 200 kDa dimeric protein) and 0.09 EU/mg endotoxin, 0.4 ng/ml DNA and 6 ng/ml host cell protein contents. Experimental vaccines contained 10 μg yeast-derived recombinant HBsAg (provided by Rhein Biotech GmbH, Düsseldorf) alone or with 3, 10, 30, 100 μg IMP321 (hLAG-3Ig). All vaccines were prepared by an unblinded pharmacist at the trial site and were administered within 1 h of mixing using a 200 μl injection volume. Each subject received three sub-cutaneous (s.c.) doses at 0, 4 and 8 weeks. The first and the third injections were done in the deltoid area of the dominant arm. The second injection was done in the deltoid area of the non dominant arm. Subjects in another comparative arm received an adult dose (1 mL) of Engerix-B^® ^(GlaxoSmithKline, Rixensart, BE) that contains 20 *μ*g of alum-absorbed yeast-derived recombinant HBsAg, which was administered intramuscularly.

### Experimental groups

Subjects were enrolled sequentially into four cohorts according to dose level of IMP321. Within cohorts, subjects were randomised to receive an experimental vaccine or control HBsAg alone in a 4:1 ratio. A total of 48 subjects were immunized according to the planned three administration schedules, 8 receiving control vaccines, 8 receiving Engerix-B^® ^and 32 receiving experimental vaccines with IMP321 (n = 8 in each group). Two subjects were prematurely discontinued from the study after the first injection and were replaced.

### Safety evaluation

All subjects who received a dose of the study drug were included in the safety evaluation (n = 50). Adverse effects were identified by clinical examination at baseline and at the following times post administration: first dose at 4 h, 48 h, one week, and 4 weeks (just prior to second dose); second dose at 4h, 48 h, one week and 4 weeks (just prior to third vaccine dose); third dose at 4 h, 48 h, one week and 4 weeks. In addition, vital signs (blood pressure and pulse rate) and oral body temperature were recorded at pre-dose, 0.5 h, 1 h, 1.5 h, 2 h and 4 h post-dosing as well as 48 h and 1 week after each injection. Laboratory tests included a complete blood count, serum chemistry, liver and renal function, and coagulation measures. Rheumatoid factors, anti-nuclear antibody titres (ANA) and anti-IMP321 antibodies were measured at baseline and weeks 12.

### Immunogenicity–humoral response

Immunogenicity results were analysed using the population which completed the study (n = 48). To assess anti-HBsAg responses, blood samples obtained at baseline and 8 and 12 weeks after the initial vaccine dosing, were allowed to clot at room temperature for 15 minutes. Samples were centrifuged at 1,500 g at about 4°C for 10 minutes and the serum was aliquoted and stored in airtight stoppered polypropylene tubes at -20°C. Sera were tested by the Abbott AUSAB-MEIA (Abbott, Abbott Park, IL, USA) and anti-HBs titres were expressed in mIU/mL based on comparison with standards defined by the World Health Organization (WHO). A protective titre was defined as ≥ 10 mIU/mL. The commercially available hepatitis B vaccine Engerix^®^-B (20 μg HBsAg adsorbed on alum) was used to ensure that our 3-months protocol schedule was able to induce antibodies in most subjects. Geometric mean of titres (GMT) was calculated using the formula 10^mean [log (Ab titers)] ^for each group at each time point. Seronegative subjects have been given the arbitrary value of 1 mIU/mL for GMT calculation.

### Data analysis

The analyses for safety and tolerability parameters were performed on all randomised subjects who received at least one dose of study medication and who had post-dose safety information (n = 50). Immunogenicity results were analysed on the population which completed the study (i.e. subjects who received 3 injections and had their post-study visit) (n = 48). Anti-HBsAg titres measured in mIU/mL were expressed as geometric mean titres (GMT) for each group. The differences between GMTs achieved at a given time point for each of the HBsAg plus IMP321 groups or the Engerix-B^® ^group were compared with the HBsAg alone group by Student's two-sided *t*-test. The proportions of subjects achieving seroconversion (anti-HBsAg ≥ 1 mIU/mL) and seroprotection (anti-HBsAg ≥ 10 mIU/mL) were compared in the combined IMP321 groups and in the Engerix-B^® ^group versus the control HBsAg alone group.

### Immunogenicity–cellular responses

#### Isolation of PBMCs

Blood was collected from healthy volunteers and from subjects included in the clinical trial at baseline and on Day 29, 36, 57 and 85 in heparin lithium tubes (BD Vacutainer™, San Jose, CA). Peripheral blood mononuclear cells (PBMCs) were immediately isolated by gradient density (Ficoll-Paque PLUS™, Amersham, Uppsala, Sweden) using LeucoSep tubes (Greiner Bio-one, Frickenhausen, Germany) resuspended in fetal calf serum (FCS, Hyclone, Logan, UT, USA) containing 10 % DMSO (Sigma Aldrich, Saint Louis, MO), slowly chilled down to -80°C (1°C/min) and cryopreserved in liquid nitrogen until analysis.

### Ex vivo stimulation of PBMC and intra-cellular staining

Before evaluating HBsAg-specific T cell responses to follow the efficacy of the immunization protocol, validation experiments were performed on four PBMCs samples collected from volunteers who had been previously immunized with commercial hepatitis B vaccine. PBMCs were thawed and stimulated using a set of 22 20-mers peptides (overlapping by 11 aa) that span the entire HBsAg protein sequence (1 μM of each peptide) or cultured with the vehicle (DMSO), in the presence of FastImmune CD28/CD49d costimulation cocktail (BD Biosciences) for 18 h and in the presence of brefeldin A (BD Biosciences) for the last 16 h. In another series of experiments, PBMC samples from three other donors were stimulated with a cytomegalovirus (CMV) pp65 peptides pool (1.75 μg/ml, BD Biosciences) or Staphylococcus Enterotoxin B (SEB, 1 μg/ml, Sigma Aldrich) in the same conditions. PBMCs unstimulated or stimulated with peptides or SEB were fixed, permeabilised using CytoFix/CytoPerm, stained with fluorochrome-conjugated CD3-PerCP-Cy5.5, CD4-PE-Cy7, CD8-APC-Cy7, IFN-γ-FITC, TNF-α-APC and IL-2-PE antibodies and extensively washed with PermWash buffer (all from BD Biosciences). Cells were then analysed using a 6-colour FACSCanto flow cytometer (BD Biosciences) to determine the percentage of CD3^+^CD4^+ ^and CD3^+^CD8^+ ^cells expressing IFN-γ, TNF-α and/or IL-2. The percentage of cells expressing cytokines in unstimulated conditions was subtracted from the percentage of cells obtained after peptide stimulation. Following completion of the protocol, a series of samples grouping the whole kinetics for each individual included in the clinical trial were thawed and analysed after 18 h of ex-vivo restimulation using the same set of HBsAg peptides. Cells were fixed, permeabilised and stained as above. A very large number of PBMCs were analysed (as an average 0.9 × 10^6 ^cells) by flow cytometry to secure the validity of small percentages and/or differences. Results following FACS analysis were defined as the difference in response to HBsAg-peptides at D29, D36, D57 or D85 versus D1. The confidence interval depended on the numbers of relevant events (CD3^+^CD4^+ ^or CD3^+^CD8^+ ^events) collected in each sample, the amount of background stimulation at D1 and difference between D1 and D29, D36, D57 or D85 time points. This difference was significant with a power of 90 % (p < 0.05) if the number of CD4^+^or CD8^+ ^cells collected was larger than calculated CD4^+ ^or CD8^+ ^events using the formula:

2×((D÷100+D1÷100)÷2)×(1−((D÷100+D1÷100)÷2)×8.6)(D÷100−D1÷100)2
 MathType@MTEF@5@5@+=feaafiart1ev1aaatCvAUfKttLearuWrP9MDH5MBPbIqV92AaeXatLxBI9gBaebbnrfifHhDYfgasaacH8akY=wiFfYdH8Gipec8Eeeu0xXdbba9frFj0=OqFfea0dXdd9vqai=hGuQ8kuc9pgc9s8qqaq=dirpe0xb9q8qiLsFr0=vr0=vr0dc8meaabaqaciaacaGaaeqabaqabeGadaaakeaadaWcaaqaaiabikdaYiabgEna0kabcIcaOiabcIcaOiabbseaejabgEpa4kabigdaXiabicdaWiabicdaWiabgUcaRiabbseaejabigdaXiabgEpa4kabigdaXiabicdaWiabicdaWiabcMcaPiabgEpa4kabikdaYiabcMcaPiabgEna0kabcIcaOiabigdaXiabgkHiTiabcIcaOiabcIcaOiabbseaejabgEpa4kabigdaXiabicdaWiabicdaWiabgUcaRiabbseaejabigdaXiabgEpa4kabigdaXiabicdaWiabicdaWiabcMcaPiabgEpa4kabikdaYiabcMcaPiabgEna0kabiIda4iabc6caUiabiAda2iabcMcaPaqaaiabcIcaOiabbseaejabgEpa4kabigdaXiabicdaWiabicdaWiabgkHiTiabbseaejabigdaXiabgEpa4kabigdaXiabicdaWiabicdaWiabcMcaPmaaCaaaleqabaGaeGOmaidaaaaaaaa@7456@

where D is the percentage of CD3^+ ^CD4^+ ^or CD3^+^CD8^+ ^cells expressing at least one cytokine on D29, D36, D57 or D85 upon stimulation and D1, the percentage of CD3^+ ^CD4^+ ^or CD3^+^CD8^+ ^cells expressing at least one cytokine on D1.

### Binding of HBsAg-specific pentamers

After completion of the protocol, PBMC harvested from a HLA-A2^+ ^donor on D1, D57 and D85 were thawed and cultured with two HLA-A2-restricted HBsAg peptides (GLSPTVWLSV and WLSLLVPFV, 1 μM each) in the presence of IL-2 (20 IU/ml) for 10 days. Fresh autologuous PBMC were loaded with the two peptides and added to the culture for additional 10 days. Fresh IL-2 was added every two days during the two rounds of stimulation. Cells were then incubated with the two HBsAg peptides/HLA-A2 pentamers (HLA-A*0201) conjugated to PE, washed, stained with CD3-PerCP-Cy5.5, CD4-APC-Cy7, CD8-FITC, CD14-APC antibodies and analysed by flow cytometry. After exclusion of CD14^+ ^monocytes, the binding of pentamers on CD3^+^CD8^+ ^cells was determined.

## Results

### Population characteristics

This study was conducted between May 2005 and December 2005. A total of 113 subjects were screened, of which 50 were enrolled and received at least one dose of vaccine. Baseline characteristics and demographics were evenly distributed among the six cohorts, with the exception of age in the HBsAg plus 10 μg IMP321 group (Table 1). All but two subjects completed the study. One subject in the HBsAg + 3 μg IMP321 and one subject in the HBsAg + 10 μg IMP321 withdrew from the study after the first immunization for personal reasons. They were replaced by 2 other subjects.

**Table 1:** Patient Characteristics (Intent-To-Treat Population)

Parameter	**Engerix^®^-B**	**HBsAg alone**	**HBsAg + 3 μg IMP321**	**HBsAg + 10 μg IMP321**	**HBsAg + 30 μg IMP321**	**HBsAg + 100 μg IMP321**
Number enrolled	8	8	9	9	8	8
Number completed	8	8	8	8	8	8
Age (years)^a ^Mean ± SD	32.1 ± 11.2	41.0 ± 11.4	31.4 ± 8.0	29.0 ± 9.2*	37.3 ± 9.7	35.9 ± 7.4
Gender^b^						
Male	7	7	9	9	8	8
Female	1	1	0	0	0	0
Race^b^						
Caucasian	7	6	6	5	6	7
Black	1	1	1	2	2	1
Asian	0	0	1	1	0	0
Other	0	1	1	1	0	0

^a ^Student's t-test. * p < 0.05 compared to HBsAg alone control group.

^b ^Chi-square. P >0.05 compared to HBsAg alone control group.

### Safety and tolerance

Overall, IMP321 plus HBsAg was characterised by a good tolerability profile at the four doses tested. A lower incidence of subjects experiencing AEs was reported after injection of IMP321 plus HBsAg (38 %) or HBsAg alone (25 %) than after injection of Engerix^®^-B (62.5 %). The most common observed non-serious adverse events included local injection site pain (4/35) and erythema (2/35), as well as systemic symptoms such as nausea (2/35) and headache (5/35) (see Table 2). Injection site pains and erythema were considered certainly related to the study drugs, whereas nausea and headache were considered possibly related. Most of these AEs were of mild to moderate intensity and resolved without any corrective treatment. Following vaccine injection, oral temperature, blood pressure, and pulse rate remained stable from baseline to hour 4, as well as on day 3 and day 8 post-dosing (data not shown). One subject from the HBsAg plus 100 μg IMP321 group developed a pruritus and a papular rash 2 hours after the first injection, which could be indicative of an allergic reaction; the symptoms were transient, not reproduced after the following injections and no medical or pharmacological intervention was required.

**Table 2:** Frequency of AEs reported during the study (Intent-To-Treat Population).

	**Engerix^®^-B (N = 8)**			**HBsAg alone (N = 8)**			**HbsAg + IMP321 3μg (N = 9)**			**HBsAg+ IMP321 10 μg (N = 9)**			**HBsAg+ IMP321 30μg (N = 8)**			**HBsAg+ IMP321 100μg (N = 8)**		
	**n**	**%**	**AE**	**n**	**%**	**AE**	**n**	**%**	**AE**	**n**	**%**	AE	**n**	**%**	**AE**	**n**	**%**	AE

**Total**	**5**	**62.5**	**5**	**2**	**25.0**	**4**	**5**	**55.6**	**8**	**2**	**22.2**	**4**	**2**	**25.0**	**3**	**4**	**50.0**	**11**

Aphthous Stomatitis	.	.	.	.	.	.	1	11.1	1	.	.	.	.	.	.	.	.	.
Diarrhoea	.	.	.	.	.	.	1	11.1	1	.	.	.	.	.	.	.	.	.
Dyspepsia	.	.	.	.	.	.	.	.	.	.	.	.	.	.	.	1	12.5	1
Nausea	1	12.5	1	.	.	.	1	11.1	1	.	.	.	.	.	.	.	.	.
Asthenia	2	25.0	2	.	.	.	.	.	.	.	.	.	.	.	.	1	12.5	1
Influenza Like Illness	.	.	.	1	12.5	1	.	.	.	.	.	.	.	.	.	.	.	.
Injection Site Erythema	.	.	.	.	.	.	.	.	.	1	11.1	1	1	12.5	1	.	.	.
Injection Site Haemorrhage	.	.	.	.	.	.	1	11.1	1	.	.	.	1	12.5	1	.	.	.
Injection Site Induration	1	12.5	1	.	.	.	.	.	.	.	.	.	.	.	.	.	.	.
Injection Site Pain	1	12.5	1	1	12.5	1	1	11.1	1	.	.	.	.	.	.	1	12.5	1
Localised Oedema	.	.	.	.	.	.	1	11.1	1	.	.	.	.	.	.	.	.	.
Herpes Simplex	.	.	.	.	.	.	1	11.1	1	.	.	.	.	.	.	.	.	.
Sinusitis	.	.	.	1	12.5	1	.	.	.	.	.	.	.	.	.	.	.	.
Urinary Tract Infection	.	.	.	.	.	.	1	11.1	1	.	.	.	.	.	.	.	.	.
Contusion	.	.	.	.	.	.	.	.	.	.	.	.	.	.	.	1	12.5	2
Back pain	.	.	.	.	.	.	.	.	.	.	.	.	.	.	.	1	12.5	1
Myalgia	.	.	.	.	.	.	.	.	.	1	11.1	1.	.	.	.	.	.	.
Headache	.	.	.	.	.	.	.	.	.	2	22.2	2	.	.	.	2	25.0	3
Erythema	.	.	.	.	.	.	.	.	.	.	.	.	1	12.5	1	.	.	.
Pruritus	.	.	.	.	.	.	.	.	.	.	.	.	.	.	.	1	12.5	1
Psoriasis	.	.	.	1	12.5	1	.	.	.	.	.	.	.	.	.	.	.	.
Rash Papular	.	.	.	.	.	.	.	.	.	.	.	.	.	.	.	1	12.5	1

There were no consistent or dose-related changes in biochemical haematological or rheumatological measures (data not shown). Moreover, antibodies to IMP321 were not detected in sera collected from subjects on D29, D36, D57 and D85 (not shown). Altogether, these data show that the injections of IMP321 were well tolerated with few reported non serious AEs and no sign of induced autoimmunity.

### Vaccine immunogenicity

#### Hepatitis B antibody titres

In our screening procedure, more than 40 % of volunteers were rejected before enrolment because of HBsAg titers above the 10 IU/mL cut-off. Following completion of the study, all sera samples were tested in a GLP laboratory to quantify titres against a WHO standard and 3 out of 48 volunteers turn not to be naïve individuals because of low HBsAg titres at Day 1 (seeTable 3). For all subsequent analyses on HBsAg antibody titres, only naïve individuals were taken into account.

**Table 3:** HBsAg antibody responses

Subject	W0	W8	W12	Subject	W0	W8	W12
**Engerix^® ^-B**				**HBsAg + IMP321 10 μg**			
003	0	**178**	**2253**	013	0	0	1
008	0	**2589**	**7055**	015	0	0	4
014	0	0	**54**	017	0	0	0
023	0	4	**442**	018	0	2	**28**
026	0	**3054**	**3156**	019	9	**504**	**609**
032	0	0	**28**	020	0	0	0
040	0	9	**164**	1021	0	0	0
043	0	2	**17**	022	0	0	0
# Seroconverted^a^	0	6 (75%)	8 (100%)	# Seroconverted^a^	0	1 (14.2%)	3 (42.8%)
# Seroprotected^a^	0	3 (37.5%)	8 (100%)	# Seroprotected^a^	0	0	1 (14.2%)
GMT^a, b^	1	23.75	313.56	GMT^a, b^	1	1.10	1.96

**HBsAg alone**				**HBsAg + IMP321 30 μg**			
006	0	0	0	025	0	0	0
012	0	0	0	027	0	0	0
016	0	0	0	029	0	0	0
024	0	0	0	030	0	0	**26**
028	0	0	7	031	0	0	0
034	0	0	0	033	0	0	4
039	0	0	2	035	2	**136**	**467**
044	1	**24**	**321**	036	0	0	0
# Seroconverted^a^	0	0	2 (28.5%)	# Seroconverted^a^	0	0	2 (28.5%)
# Seroprotected^a^	0	0	0	# Seroprotected^a^	0	0	1 (14.2%)
GMT^a, b^	1	1	1.46	GMT^a, b^	1	1	1.94

**HBsAg + IMP321 3 μg**				**HBsAg + IMP321 100 μg**			
001	0	**540**	**2229**	037	0	0	3
002	0	**35**	**51**	038	0	0	3
004	0	0	0	041	0	0	0
005	0	0	0	042	0	0	0
007	0	0	1	045	0	0	0
009	0	0	**12**	046	0	0	0
1010	0	0	1	047	0	0	0
011	0	0	5	048	0	0	0
# Seroconverted^a^	0	2 (25%)	6 (75%)	# Seroconverted^a^	0	0	2 (25%)
# Seroprotected^a^	0	2 (25%)	3 (37.5%)	# Seroprotected^a^	0	0	0
GMT^a, b^	1	3.42	7.15	GMT^a, b^	1	1	1.31

Protective titres are indicated in bold

^a ^GMT, seroconversion and seroprotection calculations were based on naïve subjects only.

^b ^Value of zero are assigned the value of 1 for the GMT calculation.

Following immunization with Engerix-B^®^, seroprotection was obtained in all subjects (100 %) after three vaccinations (Table 3), confirming the validity of our 1 and 2 months booster immunization schedule (i.e. compared to the 1 and 6 months schedule). In 5 subjects out of 8, the third immunization was necessary to obtain seroprotection. These numbers are consistent with previously published results on seroconversion following vaccination with Engerix-B^®^.

Following immunization with HBsAg alone, induction of low anti-HBsAg antibodies titres, not allowing seroprotection was observed in two out of 7 naïve subjects (28.5 %) (Table 3). It is however interesting to note that seroprotection was induced in a subject who already exhibited anti-HBsAg antibodies at low level at Day 1 (Table 3). Together, these results show that 10 μg HBsAg alone has a relatively poor immunogenic activity when not adsorbed on alum (i.e., no protection from antigen protein degradation, no long-term antigen depot effect). It is able to boost a memory response but not able to prime *de novo *naïve T cells and to induce a seroprotective antigen-specific B-cell immune response.

Addition of IMP321 to HBsAg resulted in earlier appearance of anti-HBs antibodies compared to the control HBsAg alone group. At four weeks post-second injection (Week 8), no naïve subjects in the control HBsAg alone group had detectable anti-HBsAg antibodies (see Table 3). In contrast, 2 out of 8 (25 %) naïve subjects receiving HBsAg plus 3 μg IMP321 had seroconverted four weeks after second injection. It should be noted that, even at this early time point, both IMP321 recipients who had seroconverted after the second immunization in the 3 μg group had attained seroprotective titers. Following the third immunization, 75 % of subjects in the HBsAg plus 3 μg IMP321 group showed seroconversion with a seroprotection rate of 37.5 %. Seroconversion and seroprotection rates at Week 12 were lower in the other IMP321 recipients groups with the exception of the HBsAg + 100 μg IMP321 group, but still above the rate obtained in the HBsAg alone control group. Despite a trend toward higher values in the 3 μg group, anti-HBs GMTs were not statistically significantly higher than the GMT for HBsAg alone recipients for Week 8 and 12 and this non-significance may in part be attributed to the small number of individuals per group.

Overall, these data show that IMP321 as an adjuvant to non-absorbed HBsAg, is able to induce HBsAg antibodies in 43 % of naïve individuals (i.e. 13 out of 30), with seroprotection being obtained in 2 and 5 naïve subjects following the second and the third immunization, respectively.

### Validation of intra-cellular staining after ex vivo stimulation with peptides

**Figure 1:**
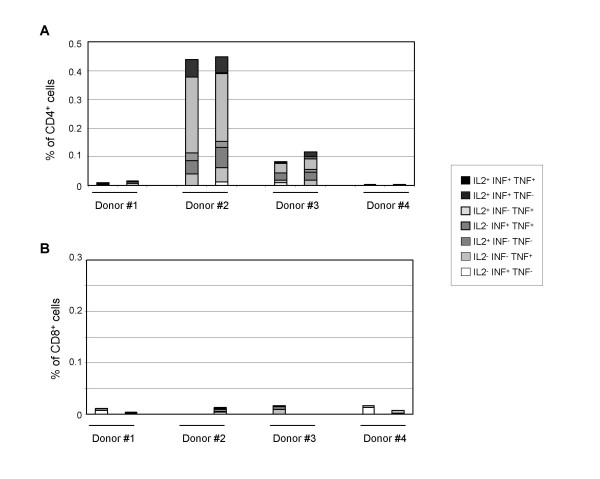
**Reproducibility of T cell responses to HbsAg**. PBMCs from 4 donors were independently purified by density gradient centrifugation and frozen by two different operators. PBMCs were then thawed and cultured with a HBsAg peptide pool or vehicle for 18 hours, in the presence of brefeldin A and the expression of IL-2, INF-γ and TNF-α in CD3^+^CD4^+ ^and CD3^+^CD8^+ ^cells was determined by specific staining and flow cytometry analysis. Background cytokine expression from unstimulated cells was subtracted from HBsAg peptide-stimulated cells. Percentages of CD4^+ ^(panel A) and CD8^+ ^(panel B) T cells either IL-2^- ^IFN-γ^+ ^TNF-α^-^, IL-2^- ^IFN-γ^-^TNF-α^+^, IL-2^+ ^IFN-γ^- ^TNF-α^-^, IL-2^- ^IFN-γ^+ ^TNF-α^+^, IL-2^+ ^IFN-γ^- ^TNF-α^+^, IL-2^+ ^IFN-γ^+ ^TNF-α^- ^or IL-2^+ ^IFN-γ^+ ^TNF-α^+ ^obtained for the four donors in two independent experiments are presented.

Before evaluating HBsAg-specific T cells response by intracellular staining to detect cytokines in T cells by flow cytometry analysis after short term *ex-vivo *stimulation with a pool of 22 HBsAg overlapping peptides (20 aa overlapping by 11), standard operation procedures were established and fixed. First, blood samples from 4 different donors previously immunized by a commercial hepatitis B vaccine were collected and PBMCs independently purified and frozen by two different operators. PBMCs were then stimulated by the HBsAg peptide pool for 18 hours in the presence of brefeldin A and stained with fluorochrome-conjugated CD3, CD4, CD8, IL-2, INF-γ and TNF-α-specific antibodies. The percentages of CD3^+^CD4^+ ^and CD3^+^CD8^+ ^cells expressing cytokines after HBsAg-stimulation obtained by the two operators are presented in Figure 1. Only two donors out of four had developed a detectable antigen-specific Th1 cytokine CD4 response after HBsAg peptide pool stimulation (Figure 1A). No CD8^+ ^T cell cytokine response was observed (Figure 1B). Similar results were obtained in the experiments performed by the two different operators.

**Figure 2:**
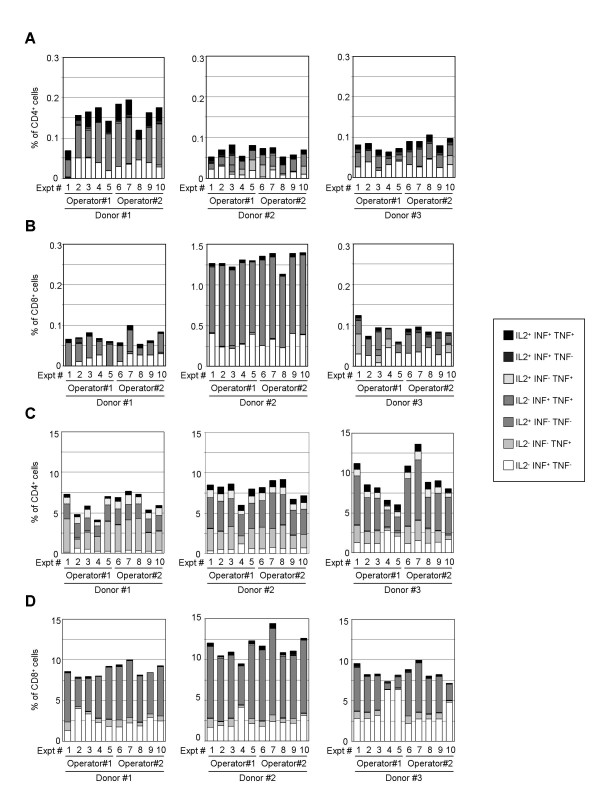
**Reproducibility of T cell responses to CMV pp65 and SEB**. Frozen PBMCs from 3 donors were independently thawed and cultured with CMV pp65 peptide pool or vehicle (panels A and B) or with SEB (panels C and D) by two operators at five different occasions. The expression of IL-2, INF-γ and TNF-α in CD3^+^CD4^+ ^and CD3^+^CD8^+ ^cells was determined by specific staining and flow cytometry analysis. Background cytokine expression from unstimulated cells was subtracted from CMV pp65 peptides-stimulated cells. Percentages of CD4^+ ^(panel A and C) and CD8^+ ^(panel B and D) T cells either IL-2^- ^IFN-γ^+ ^TNF-α^-^, IL-2^- ^IFN-γ^- ^TNF-α^+^, IL-2^+ ^IFN-γ^- ^TNF-α^-^, IL-2^- ^IFN-γ^+ ^TNF-α^+^, IL-2^+ ^IFN-γ^- ^TNF-α^+^, IL-2^+ ^IFN-γ^+ ^TNF-α^- ^or IL-2^+ ^IFN-γ^+ ^TNF-α^+ ^obtained for the three donors in ten independent experiments are presented.

Since CMV-specific CD8 responses are easily observed in normal donors, the reproducibility of the intra-cellular staining method to detect cytokine expression in CD4 and CD8 T cell subpopulations was performed after stimulation with a peptide pool spanning the sequence of CMV pp65. PBMCs from three donors were stimulated with the CMV pp65 peptides and stained to detect cytokine expression in ten independent experiments performed by two operators (Figure 2A and 2B). Control stimulation with SEB superantigen was added (Figure 2C and 2D). All three PBMC samples displayed a detectable cytokine response in both CD4 and CD8 T cell subsets after CMV pp65 stimulation. A high frequency of CMV pp65-specific Tc1 CD8^+ ^T cells was found in donor#2's PBMCs (>1 %, Figure 2B, middle panel). The means and standard deviations of the percentages of CD4^+ ^and CD8^+ ^T cells expressing these cytokines were calculated for the three donors. Inter-experiments and inter-operators coefficient variations (CV) were determined. Repeatability of the results obtained by each operator was 17 % and 14 % for both T cell populations. Overall inter-experiments and inter-operators CV were 19 % and 15 % for antigen-specific CD4 and CD8 response, respectively. Inter-experiments and inter-operators CV calculated for SEB-stimulation were 19 % and 10 % for CD4 and CD8 populations, respectively. To avoid additional variability, the monitoring of the T cell response in the clinical trial was performed by a single operator who obtained 15 % and 14 % CV on CD4 and CD8 antigen-specific responses, respectively.

### Hepatitis B-specific T cell responses

**Figure 3:**
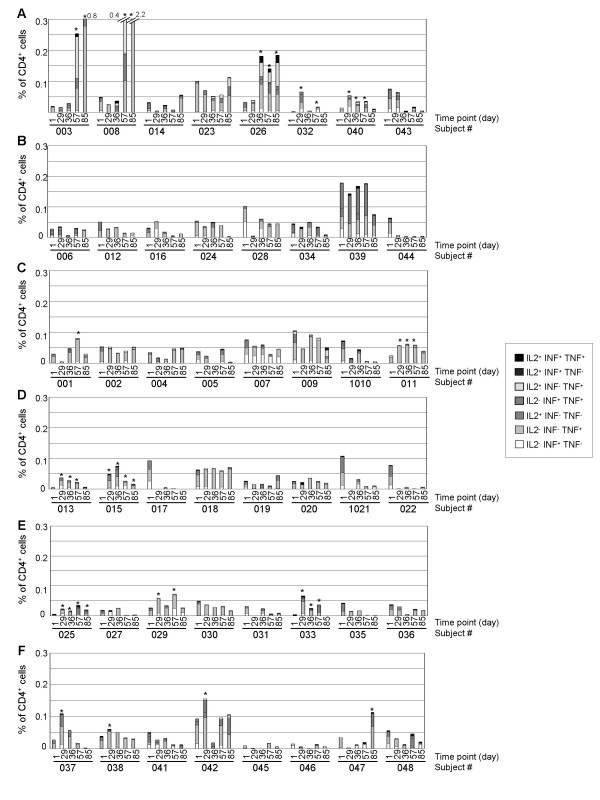
**Percentage of CD4^+ ^T cells expressing IFN-γ, TNF-α and/or IL-2 upon HBsAg-peptide stimulation**. PBMC were isolated from whole blood by density gradient and frozen. Following completion of the protocol, cells were thawed and cultured with 22 HBsAg 20-mers peptides or with vehicle for 18 hours, in the presence of brefeldin A. PBMC were then fixed, permeabilized and stained with fluorochrome-conjugated CD3, CD4, CD8, IFN-γ, TNF-α, IL-2 specific antibodies. The percentage of CD3^+^CD4^+ ^T lymphocytes expressing IFN-γ, TNF-α and/or IL-2 was determined by flow cytometry. Background cytokine expression from unstimulated cells was subtracted from HBsAg peptides-stimulated cells. Percentages of CD4^+ ^lymphocytes either IL-2^- ^IFN-γ^+ ^TNF-α^-^, IL-2^- ^IFN-γ^- ^TNF-α^+^, IL-2^+ ^IFN-γ^- ^TNF-α^-^, IL-2^- ^IFN-γ^+ ^TNF-α^+^, IL-2^+ ^IFN-γ^- ^TNF-α^+^, IL-2^+ ^IFN-γ^+ ^TNF-α^- ^or IL-2^+ ^IFN-γ^+ ^TNF-α^+ ^are presented in groups Engerix-B^® ^(panel A), HBsAg alone (panel B), HBsAg + 3 μg IMP321 (panel C), HBsAg + 10 μg IMP321 (panel D), HBsAg + 30 μg IMP321 (panel E) and HBsAg + 100 μg IMP321 (panel F) for every subject at every time point (see x-axis). Statistically significant increases (p < 0.05) are shown by an asterisk.

To investigate the T cell response to HBsAg vaccination in the different groups, PBMCs were cultured for 18 hr with the same pool of 22 HBsAg overlapping peptides and the number of antigen-specific T cells was determined by flow cytometry after IFN-γ, TNF-α, and IL-2 intracellular staining in CD3^+^CD4^+ ^and CD3^+^CD8^+ ^cells. The percentage of CD4^+ ^T cells expressing these Th1-type cytokines upon stimulation with antigenic peptides is shown in Figure 3.

**Figure 4:**
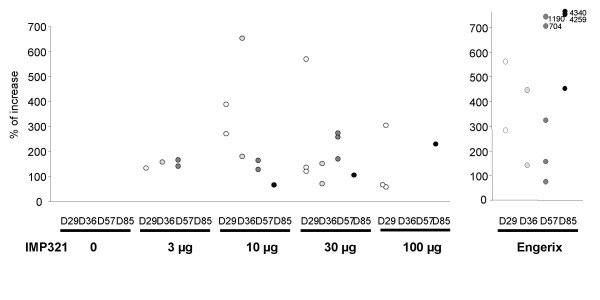
**Induction of CD4^+ ^Th1 cell response to HBsAg peptides**. Unstimulated and HBsAg peptides-stimulated PBMC were stained with fluorochrome-conjugated CD3, CD4, CD8, IFN-γ, TNF-α, IL-2 specific antibodies. The percentage of CD3^+^CD4^+ ^T lymphocytes expressing at least one cytokine was determined by flow cytometry. Background cytokine expression from unstimulated cells was subtracted from HBsAg peptides-stimulated cells and the induction of Th1 response at D29 (open circle), D36 (gray circle), D57 (dark gray circle) or D85 (closed circle) compared to D1 was calculated for each subject displaying a statistically significant increase (p < 0.05, see Figure 3) using the formula: (% of cytokines+ cells at D29 or D36 or D57 or D85)×100% of cytokines+ cells at D1−100
 MathType@MTEF@5@5@+=feaafiart1ev1aaatCvAUfKttLearuWrP9MDH5MBPbIqV92AaeXatLxBI9gBaebbnrfifHhDYfgasaacH8akY=wiFfYdH8Gipec8Eeeu0xXdbba9frFj0=OqFfea0dXdd9vqai=hGuQ8kuc9pgc9s8qqaq=dirpe0xb9q8qiLsFr0=vr0=vr0dc8meaabaqaciaacaGaaeqabaqabeGadaaakeaadaWcaaqaaiabcIcaOiabcwcaLiabbccaGiabb+gaVjabbAgaMjabbccaGiabbogaJjabbMha5jabbsha0jabb+gaVjabbUgaRjabbMgaPjabb6gaUjabbwgaLjabbohaZnaaCaaaleqabaGaey4kaScaaOGaeeiiaaIaee4yamMaeeyzauMaeeiBaWMaeeiBaWMaee4CamNaeeiiaaIaeeyyaeMaeeiDaqNaeeiiaaIaeeiraqKaeGOmaiJaeGyoaKJaeeiiaaIaee4Ba8MaeeOCaiNaeeiiaaIaeeiraqKaeG4mamJaeGOnayJaeeiiaaIaee4Ba8MaeeOCaiNaeeiiaaIaeeiraqKaeGynauJaeG4naCJaeeiiaaIaee4Ba8MaeeOCaiNaeeiiaaIaeeiraqKaeGioaGJaeGynauJaeiykaKIaey41aqRaeGymaeJaeGimaaJaeGimaadabaGaeiyjauIaeeiiaaIaee4Ba8MaeeOzayMaeeiiaaIaee4yamMaeeyEaKNaeeiDaqNaee4Ba8Maee4AaSMaeeyAaKMaeeOBa4MaeeyzauMaee4Cam3aaWbaaSqabeaacqGHRaWkaaGccqqGGaaicqqGJbWycqqGLbqzcqqGSbaBcqqGSbaBcqqGZbWCcqqGGaaicqqGHbqycqqG0baDcqqGGaaicqqGebarcqaIXaqmaaGaeyOeI0IaeGymaeJaeGimaaJaeGimaadaaa@8F19@

Five subjects out of 8 (62.5 %) in the Engerix-B^® ^group displayed an increase in the percentage of CD4 T cell expressing cytokines upon stimulation with HBsAg peptides on D29, D36, D57 or D85 compared to D1 (Figure 3A). In contrast, no subject displayed a significant increase of the percentage of responding CD4^+ ^T cells in the HBsAg alone group (Figure 3B), indicating that the antigen alone was unable to induce a detectable CD4^+ ^T cells response, even in one antigen-experienced subject (#044) who became seroprotected after vaccination (Table 3). In groups receiving IMP321, an increase in the frequency of specific CD4^+ ^T cells producing Th1-type cytokines was observed in 2 subjects (25 %) in the 3 and 10 μg groups (Figure 3C and 3D), 3 subjects in the 30 μg group (37.5 %, Figure 3E) and 4 in the 100 μg group (50 %, Figure 3F). Thus exposure to IMP321 is associated with the induction of a detectable antigen-specific Th1 CD4 cell response in 25 to 50 % of the subjects compared to 0 % in the absence of IMP321. It is to note that none of the three antigen-experienced subjects pre-vaccination developed a CD4 Th1 response even if Ab titres were boosted (see above). Importantly, the kinetics of circulating CD4^+ ^T cells response was heterogeneous in responding subjects. While the T cell response kept increasing over time for three subjects (two in the Engerix^®^-B group, one in the IMP321 100 μg group), repeating injections resulted in all the others in a decrease of Th1 CD4 T cell frequencies in blood.

In addition to the increased number of subjects displaying a Th1 CD4 cell response in the IMP321 groups compared to HBsAg alone, the magnitude of the Th1 response was also determined. Figure 4 shows the fold increase percentages of CD4^+ ^T cells expressing at least one cytokine upon HBsAg stimulation on D29, D36, D57 and D85 versus D1 for each responder. In the subjects who displayed a significant increase, CD4^+ ^T cell response seems to be more intense in cohorts 10 or 30 μg IMP321 compared to 3 μg and 100 μg. Strikingly, at D29, i.e. after only a single injection, the response in IMP321 recipients was as intense as the one observed in the Engerix-B^® ^group. As mentioned above, the intensity of the response at D85 seems to decrease compared to other time points in every groups, whereas 2 subjects in the Engerix-B^® ^group displayed a continuous increase in the intensity of the response.

For the early time point D29, IMP321 plus 10 μg HBsAg is as efficient at inducing the CD4^+ ^T cell responses as an established commercial vaccine incorporating 20 μg HBsAg adsorbed onto alum, a process known to protect HBsAg from degradation with, in addition, a depot effect for long lasting antigen release. For later time points, the mix of IMP321 plus HBsAg is unable to induce further increases (in contrast to Engerix-B^® ^in two individuals).

**Figure 5:**
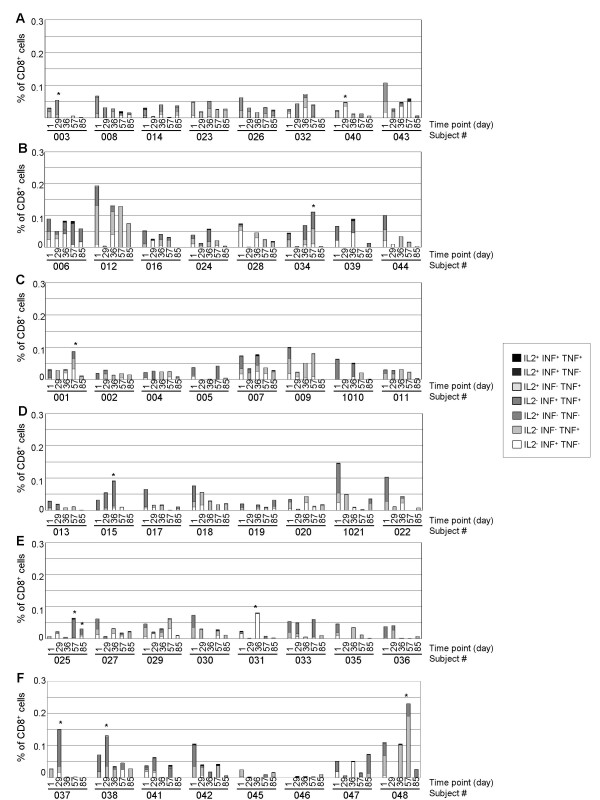
**Percentage of CD8^+ ^T cells expressing IFN-γ, TNF-α and/or IL-2 upon HBsAg-peptide stimulation**. PBMC were stimulated with HBsAg peptides and stained as described in the legend to Figure 3. The percentage of CD3^+^CD8^+ ^T lymphocytes expressing IFN-γ, TNF-α and/or IL-2 was determined by flow cytometry. Background cytokine expression from unstimulated cells was subtracted from HBsAg peptides-stimulated cells. Percentages of CD8^+ ^lymphocytes either IL-2^- ^IFN-γ^+ ^TNF-α^-^, IL-2^-^IFN-γ^- ^TNF-α^+^, IL-2^+ ^IFN-γ^-^TNF-α^-^, IL-2^- ^IFN-γ^+ ^TNF-α^+^, IL-2^+ ^IFN-γ^- ^TNF-α^+^, IL-2^+ ^IFN-γ^+ ^TNF-α^- ^or IL-2^+ ^IFN-γ^+ ^TNF-α^+ ^are presented in groups Engerix-B^® ^(panel A), HBsAg alone (panel B), HBsAg + 3 μg IMP321 (panel C), HBsAg + 10 μg IMP321 (panel D), HBsAg + 30 μg IMP321 (panel E) and HBsAg + 100 μg IMP321 (panel F) for every subject at every time point (see x-axis). Statistically significant increase (p < 0.05) are shown by an asterisk.

**Figure 6:**
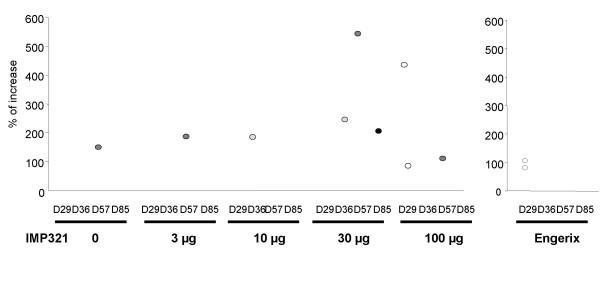
**Induction of CD8^+ ^Tc1 cell responses to HBsAg peptides**. Unstimulated and HBsAg peptides-stimulated PBMC were stained with fluorochrome-conjugated CD3, CD4, CD8, IFN-γ, TNF-α, IL-2 specific antibodies. The percentage of CD3^+^CD8^+ ^T lymphocytes expressing at least one cytokine was determined by flow cytometry. Background cytokine expression from unstimulated cells was subtracted from HBsAg peptides-stimulated cells and the induction of Tc1 response at D29 (open circle), D36 (gray circle), D57 (dark gray circle) or D85 (closed circle) compared to D1 was calculated for each subject displaying a statistically significant increase (p < 0.05, see Figure 5) using the formula: (% of cytokines+ cells at D29 or D36 or D57 or D85)×100% of cytokines+ cells at D1−100
 MathType@MTEF@5@5@+=feaafiart1ev1aaatCvAUfKttLearuWrP9MDH5MBPbIqV92AaeXatLxBI9gBaebbnrfifHhDYfgasaacH8akY=wiFfYdH8Gipec8Eeeu0xXdbba9frFj0=OqFfea0dXdd9vqai=hGuQ8kuc9pgc9s8qqaq=dirpe0xb9q8qiLsFr0=vr0=vr0dc8meaabaqaciaacaGaaeqabaqabeGadaaakeaadaWcaaqaaiabcIcaOiabcwcaLiabbccaGiabb+gaVjabbAgaMjabbccaGiabbogaJjabbMha5jabbsha0jabb+gaVjabbUgaRjabbMgaPjabb6gaUjabbwgaLjabbohaZnaaCaaaleqabaGaey4kaScaaOGaeeiiaaIaee4yamMaeeyzauMaeeiBaWMaeeiBaWMaee4CamNaeeiiaaIaeeyyaeMaeeiDaqNaeeiiaaIaeeiraqKaeGOmaiJaeGyoaKJaeeiiaaIaee4Ba8MaeeOCaiNaeeiiaaIaeeiraqKaeG4mamJaeGOnayJaeeiiaaIaee4Ba8MaeeOCaiNaeeiiaaIaeeiraqKaeGynauJaeG4naCJaeeiiaaIaee4Ba8MaeeOCaiNaeeiiaaIaeeiraqKaeGioaGJaeGynauJaeiykaKIaey41aqRaeGymaeJaeGimaaJaeGimaadabaGaeiyjauIaeeiiaaIaee4Ba8MaeeOzayMaeeiiaaIaee4yamMaeeyEaKNaeeiDaqNaee4Ba8Maee4AaSMaeeyAaKMaeeOBa4MaeeyzauMaee4Cam3aaWbaaSqabeaacqGHRaWkaaGccqqGGaaicqqGJbWycqqGLbqzcqqGSbaBcqqGSbaBcqqGZbWCcqqGGaaicqqGHbqycqqG0baDcqqGGaaicqqGebarcqaIXaqmaaGaeyOeI0IaeGymaeJaeGimaaJaeGimaadaaa@8F19@

Concerning the production of Tc1 cytokines by CD8^+ ^T cells after *ex vivo *stimulation with HBsAg peptides, only a few subjects developed a higher frequency of CD8^+ ^T cells at least at one kinetics time point compared to baseline. In contrast to the strong response of the CD4^+ ^T cells of most of the subjects in the Engerix-B^® ^group, a significant but slight CD8^+ ^T cell response was observed in only two subjects (25 %) one month after the first injection of the vaccine (Figure 5A). In HBsAg alone and 3 or 10 μg IMP321 groups, one volunteer out of 8 (12.5 %) displayed an significant increase in the percentage of CD8 T cells expressing IFN-γ, TNF-α or IL-2 (Figure 5B, C, and 5D). In the 30 or 100 μg IMP321 groups, respectively, 2 (25 %) and 3 (37.5 %) subjects exhibited an increase in the percentage of responding CD8^+ ^T cells (Figure 5E and 5F). Regarding the magnitude of the Tc1 response in subjects exhibiting a significant increase in CD8^+ ^T cells, one subject both in the 30 μg and the 100 μg groups displayed an intense response (Figure 6).

**Figure 7:**
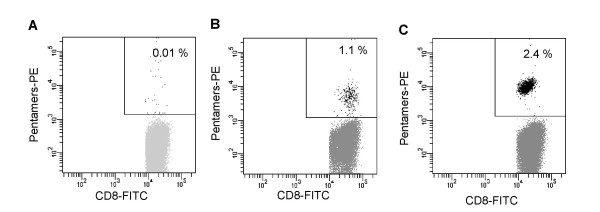
**Binding of HBsAg-specific pentamers on CD8^+ ^T cells in a subject injected with 100 μg IMP321**. PBMC from a naïve HLA-A2^+ ^volunteer collected at baseline, on D57 and D85 were thawed and cultured with two HLA-A2-restricted HBsAg peptides in the presence of IL-2 for 10 days. Fresh autologous PBMC loaded with the two peptides were added to the culture for the second round of stimulation, and the culture was maintained for another 10 days. Cells were then incubated with two HBsAg peptides/HLA-A2 pentamers, washed and stained with CD3, CD4, CD8, CD14 specific antibodies. Percentage of CD3^+^CD8^+ ^cells stained by the pentamers was analysed by flow cytometry after exclusion of CD14^+^cells. Dot plots showing the binding of HBsAg-pentamers on CD8^+ ^T cells on D1 (Panel A) and D57 (Panel B) after two rounds of stimulation and on D85 after one round of stimulation are presented.

As for the CD4^+ ^T cells response, the CD8 cells response assessed in our short term *ex vivo *assay decreased after repeated immunization. PBMCs from one subject in the 100 μg IMP321 group (#037, HLA-A2^+^) who displayed a detectable Th1 and a Tc1 response after only one immunization (D29) and no response after the second and the third immunization, were cultured for one or two rounds of *in vitro *stimulation with two HLA-A2-restricted peptides. After amplification of the specific T cells with the peptides, the number of CD8^+ ^T cells bearing a TCR recognizing one of these two peptides presented on HLA-A2 molecules was determined by staining with pentamers loaded with the peptides (Figure 7). The percentage of specific CD8^+ ^T cells detected with pentamers was higher in PBMC from D57 and D85 compared to D1. Moreover, 10-day stimulation was sufficient to induce 2.4% of HBsAg specific CD8^+ ^T cells from PBMC collected after the third immunization. Thus, even if no detectable cytokine positive T cells were detected in our short term *ex vivo *assay, specific CD8^+ ^T cells with vigorous proliferative potential were still present in low numbers (i.e. below the detection ICS assay threshold of 0.01 % for CD8 cells) after repeated injection of IMP321.

## Discussion

To study the efficacy of IMP321 as an immunopotentiator in man, we have used state-of-the-art immunomonitoring techniques (i.e. direct *ex vivo *6 colour FACS analysis of antigen-specific CD4 or CD8 cells producing IL-2, IFNγ or TNF-α as detected by intra-cellular staining). Intra-cellular staining methods allow the phenotyping of the cytokines-producing cells with good reproducibility (Figures 1 and 2) without any cell sorting as required with standard Elispot method. Moreover, at a single cell level, several cytokines can be detected, which is not possible using standard Elispot, allowing a better coverage of the heterogeneous subsets being induced (e.g. IL-2 for the memory phenotype).

In the present study, both high levels of Th1 CD4 and Tc1 CD8 T cells (i.e. more than 0.1 % in the corresponding subset) have been detected in some individuals immunized with 10 or 30 μg IMP321 and 10 μg HBsAg without alum even though the HBsAg is probably at a suboptimal dose due to the absence of protection from antigen degradation or of a depot effect from the alum. The same result has also been obtained in a previous phase I trial testing 10 and 30 μg IMP321 and a flu vaccine (manuscript submitted).

Compared to alum, injecting IMP321 with 10 μg of HBsAg was equivalent in terms of CD4^+ ^T cell responses to injecting 20 μg of alum-absorbed HBsAg (i.e. Engerix-B^®^) after the first injection, even though there was neither antigen protection nor any antigen depot effect in the former condition. However, there was no consistent build-up of either CD4 or CD8 responses after the second and third injections. Indeed, we observed a decrease of circulating responding T cells after D29 in most of the subjects. It remains possible that T cells which became antigen-experienced after the first immunization home into lymphoid organs following subsequent immunizations. Indeed, very few antigen-specific CD8 cells (i.e. undetectable without *in vitro *amplification) remained in the blood at D57 or D85, in line with previous observations showing a compartmentalization to lymphoid tissues [12].

Despite more than 10 years of research, TLR agonists have not succeeded in showing good T cell response adjuvanticity ratio *in vivo*. For instance, CpG ODN have been shown to increase HBsAg Ig titers and Ab affinity, but the induction of HBsAg-specific T cells could not be detected in direct *ex vivo *assays (i.e. without any bias induced by *in vitro *lymphocyte proliferation) in immunized healthy individuals co-injected with 3 mg ISS [13] or with 1 mg CpG ODN [14], respectively. Similarly, cohorts of 30 healthy individuals immunized with a full-dose flu vaccine (Fluarix^®^) plus 1 mg CpG 7909 did not reveal an increased cellular response induced by CpG [15]. Therefore the potential for TLR9 ligands to enhance CTL responses in humans has thus far not been shown, except for one clinical study investigating a vaccination of melanoma patients with a Melan/MART-1 peptide plus 0.5 mg CpG emulsified in Montanide^® ^[16].

## Conclusion

In conclusion, IMP321 as an adjuvant to HBsAg was well-tolerated and enhanced T cell response vaccine immunogenicity (i.e. induced both CD4 Th1 and CD8 Tc1 antigen-specific T cells). Its ability to orientate the immune response to Th1/Tc1 was confirmed by the greater increase in the cellular than the humoral response as would be expected for a therapeutic vaccine adjuvant. Future clinical studies are underway to assess the potential of such non-inflammatory non-TLR ligands used alone or as adjuvants for therapeutic vaccines.

## Competing interests

The authors are employees of Immutep S.A. and F. Triebel holds equity interests in Immutep S.A.

## Authors' contributions

CB supervised the pharmacodynamics part of the study, was involved in data analysis and in the drafting of the manuscript. CG performed immunoassays, data acquisition and analysis. MM carried out blood cells isolation and stimulation. GP was involved in the coordination of the study and in the drafting of the manuscript. FT conceived and supervised the study and finalized the manuscript. All authors read and approved the final manuscript.
